# How to return? experiences of patients in working age after first Ischaemic stroke: an interpretative phenomenological analysis of patient´s perspective at 12 – 24 months post-stroke

**DOI:** 10.1080/17482631.2024.2398249

**Published:** 2024-09-04

**Authors:** Šárka Šaňáková, Elena Gurková, Lenka Štureková, Daniela Bartoníčková, Lenka Machálková, Lenka Mazalová

**Affiliations:** Department of Nursing, Faculty of Health Sciences, Palacký University Olomouc, Olomouc, Czech Republic

**Keywords:** Ischaemic stroke, interpretative phenomenological analysis, patient experience, young adult, working age

## Abstract

**Background:**

Limited evidence of young adult patient-reported outcomes and experiences after ischaemic stroke has been conducted.

**Aim:**

To investigate the meaning of the lived experiences of stroke patients in working age 12–24 months after their first IS.

**Material and Methods:**

The exploratory qualitative study used an interpretative phenomenological analysis (IPA) design. Nine ischaemic stroke patients (with age ranges from 41 to 50 years) took part in semi-structured qualitative interviews.

**Results:**

Even with mild residual neurological deficit, IS negatively impacted the quality of life daily and social life. Six subthemes and three interconnected group experiential themes were generated: (i) *From confusion to understanding (ii) Triggers for rebuilding; and (iii) Challenges and benefits.*

**Conclusion:**

The study highlights the current gaps and limitations in supporting the needs of stroke patients in working age in long-term post-stroke care. The findings are crucial for healthcare professionals to develop improved age- and mild- impairment-appropriate strategies or tailor self-management interventions for stroke patients of working age.

**ClinicalTrials.gov**: NCT04839887.

## Introduction

Ischaemic stroke (IS) is a leading cause of long-term disability and death worldwide, predominantly affecting the elderly population (World Health Organization, 2020). However, emerging evidence suggests that stroke incidence among young adults, defined as individuals between 18 and 50 years of age, is on the rise, presenting unique challenges and consequences for this age group (Béjot et al., 2016; Putaala, 2016; Yahya et al., 2020). In the United States, IS in young adults accounts for approximately 10–15% of all reported strokes (Benjamin et al., 2017). The experiences of young, working-age adults after stroke encompass various physical, cognitive, emotional, and social dimensions that significantly impact their quality of life and functional independence. Adults of working age recovering from IS have significantly different experiences of family dynamics, work, and societal pressures compared to older survivors of IS (Maaijwee et al., 2014; Synhaeve et al., 2015). Young adults coping with IS encounter these obstacles during a crucial phase of life when they aim to build families, achieve notable professional progress, and sustain vibrant social connections (Synhaeve et al., 2015). For this reason, it is the unique experiences of young adults after IS that need to be addressed.

Although several reviews are looking at this issue, they have focused on older patients—in the 45–65 age group (Ford et al., 2021; Guo et al., 2021; Harris & Prvu Bettger, 2018; Holloway et al., 2022). These recent research syntheses provide evidence on specific post-IS topics, such as parenting (Harris & Prvu Bettger, 2018), close personal relationships (Ford et al., 2021), social consequences, returning to work (Daniel et al., 2009), unmet needs (Guo et al., 2021), or rehabilitation needs and experiences (Holloway et al., 2022). However, studies included in these reviews were heterogeneous in terms of stroke type or definition of young stroke patients, with the upper age limit ranging from 45 to 65 years. Only seven qualitative studies (Hanney, 2012; Hutton & Ownsworth, 2019; Immenschuh, 2003; Kuluski et al., 2014; Leahy et al., 2016; Opoku et al., 2020; Wolfenden & Grace, 2015) explicitly focused on the experiences of young IS survivors. These studies highlighted themes of disruption of self and self-identity, re-establishment of one’s new self, social disruption, re-establishment of one’s social role, disruption of close personal relationships, and relationships help (capturing the positives which relationships brought to stroke survivors’ lives).

Only two available reviews specifically addressed young adults after stroke (Gurková et al., 2023; Lawrence, 2010). The former (Lawrence, 2010) identified the following categories: disorientation; disrupted sense of self; and roles and relationships. The effects of stroke are often “invisible” but can have a substantial impact on social participation. This includes challenges in returning to work and maintaining an active social life. Young adults, in particular, experience both similarities and differences in their post-stroke reality, which can profoundly affect their relationships. However, the explanation provided did not delve into the internal challenges faced by young adults. On the other hand, the recently published review (Gurková et al., 2023) offers not only the main categories but also strategies for coping with the changes associated with overcoming IS. At a young age, stroke may be associated with three distinct domains: disruption of self and self-identity; social disruption; and disruption of close personal relationships. The authors also describe coping strategies to deal with these disruptions: re-establishing a new self, re-establishing a social role, and relationship help. There is a need to directly and clearly define the experience of adults of working age after IS to plan quality care.

## Aim

The present study aimed to better understand the young stroke patients’ lived experience at 12–24 months after their first-ever IS. The research questions were as follows: What is the meaning of the lived experiences of young, working-age stroke patients 12–24 months after their first IS? What are the subjective accounts of working-age adults’ experiences of stroke, and how did they perceive the impact of IS on their lives 12–24 months after their first-ever IS?

## Methods

### Study design

The current exploratory qualitative study employed an interpretative phenomenological analysis (IPA) design to investigate the significance of living with young stroke. IPA is a methodology that thoroughly explores individuals’ lived experiences and their interpretation of those experiences within the framework of their personal and social environments (Smith & Nizza, 2022; Smith et al., 2021). As an experiential qualitative method, IPA is grounded in three theoretical foundations: phenomenology, hermeneutics, and idiography (Smith & Nizza, 2022). IPA is affected by the phenomenological tradition because of the exploring of a specific lived experience from a first-person perspective. This methodology is an appropriate approach for this study; using IPA allowed young adults to talk openly about their experience of IS, thus gaining a deeper understanding of the sequels after IS from the perspective of the stroke survivor. Exploring the experiences of young stroke patients has been neglected in research studies, and the existing literature tends to focus on the older population rather than younger adult stroke survivors (Holloway et al., 2022). Participants in IPA are viewed as the experts of their own lives; the researcher’s role during the semi-structured interview is to help participants access their personal world by providing open-ended questions and prompts to provide the opportunity for deeper reflection (Smith et al., 2009). The focus on the process of sense-making in IPA comes from the hermeneutic tradition. Accordingly, the interviews in IPA focus on how participants make sense of their experience. In our case, this refers to young adults’ thoughts and feelings about experiences of stroke, the psychosocial impact of stroke on adults of working age, and the identification of their coping strategies. In the analytic process, the researcher makes sense of the participant, who is making sense of their lived experience (Smith et al., 2009). Finally, IPA is strongly idiographic, and a particular interview is analysed within the context of the individual’s narratives before patterns of similarity and differences between cases are highlighted during cross-case analysis. Using IPA allows for convergence and divergence within the sample (Smith, 2017). This methodology has been used in previous research to capture the lived experience of stroke in working-age patients (Hanney, 2012; Hutton & Ownsworth, 2019; Leahy et al., 2016). In these studies, the time post-onset of stroke ranged from six months to 31 years (Hutton & Ownsworth, 2019). Therefore, this study focused on a more homogenous group of patients. Analysing the data collected using IPA allows for a thorough exploration of stroke survivors’ experiences, which was considered the most appropriate approach to achieve the study aims of exploring the lived experiences of patients of working age to understand how they perceive and make sense of stroke as a disabling life situation. IPA attempts to move away from the limitations of the biomedical model of illness, where functional status and outcomes are crucial, towards a deeper understanding of the subjective experience of illness (Hanney, 2012) and patient perspectives in the delivery of post-stroke long-term care.

### Sample

The study sample was selected purposively. Consecutive IS patients, who were enrolled in the prospective study FRAILTY (Factors Affecting the Quality of Life After Ischemic Stroke in Young Adults; ClinicalTrials.gov. Identifier: NCT04839887, registered on 9 April 20219 April 2021) during a period between 01/2023 and 05/2023 were screened for participation in this study. The FRAILTY study explores how initial stroke-related and personal factors and functional outcomes are associated with health-related quality of life in younger individuals in the first 12 months after IS. However, there is strong empirical evidence that psychosocial sequels after IS seem to persist across time. Therefore, we decided to explore a long-term perspective on the complexities underpinning the recovery and rehabilitation process. Patients at least one-year post-IS after IS were included.

Adults of working age who had suffered IS and attended a neurological outpatient clinic at a university hospital cerebrovascular centre were recruited for the study. Recruitment was focused on individuals who had undergone regular follow-up examinations and were at least one-year post-IS. The following inclusion criteria guided the selection of participants: individuals aged 18–50 years, at least one year post-IS, and consent to an interview. The exclusion criteria were as follows: transient ischaemic attack without progression to IS; cerebral infarction caused by trauma; haemorrhagic stroke; severe cognitive impairment or communication disorder hindering participation in an in-depth interview; a concomitant severe systemic illness potentially affecting the quality of life after IS. Patients with haemorrhagic stroke were excluded as their prognosis and experiences could differ from those of IS survivors. The National Institute of Health Stroke Scale (NIHSS) assessed the residual neurological deficit at enrolment. All participants scored NIHSS < 5 at the interview (no stroke symptoms or a minor stroke).

**Figure 1.**
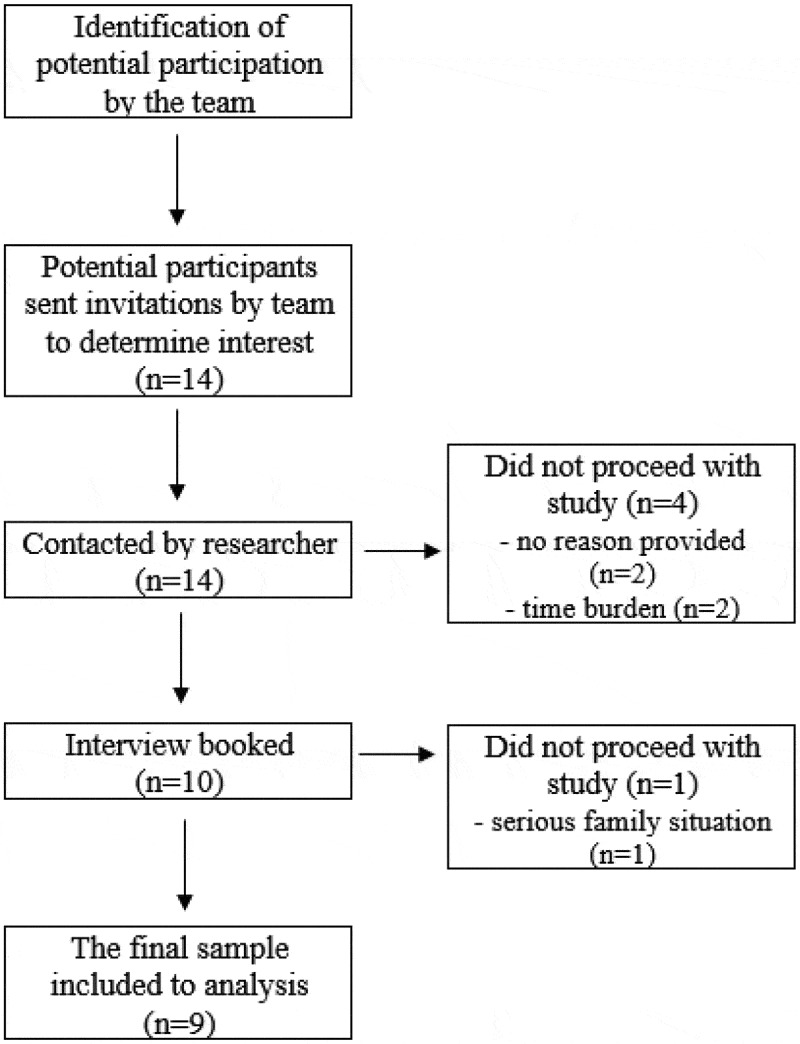
Flow chart of the recruitment process.

An experienced neurologist and clinical nurse specialists at the stroke centre acted as gatekeepers for this study. They identified potential participants, providing them with invitation letters and information sheets explaining the survey. The gatekeeper then gave the researchers the contact details of anyone who expressed an interest in taking part. An experienced neurologist screened and selected eligible patients (*n* = 14) according to inclusion criteria, and the research team contacted eligible patients. The research team approached eligible patients (*n* = 14). Finally, nine individuals (four women and five men) agreed to participate (Table I). Figure 1 outlines recruitment into the study. The final number depended on the willingness of patients to participate in the study. There was no previous relationship between the participants and the interviewers.

**Table I.** The participants’ characteristics.

Participant (interview)	Gender	Age	Education	Occupation	Marital status	Measure time (time of assessment after stroke – months)	NIHSS score at hospital discharge	NIHSS score at the time of the interview
John	male	50	secondary (vocational)	manual worker	single	12	1	1
Nina	female	44	secondary (vocational)	club keeper	single, live with a partner	24	7	3
Jack	male	48	secondary (vocational)	manual worker	married	12	1	0
Oliver	male	45	secondary (vocational)	machine operator	married	24	0	0
Emily	female	43	secondary (vocational)	unemployed	married	12	0	0
George	male	43	secondary (school-leaving examination)	project architect	married	14	9	3
Sophie	female	50	tertiary	teacher	divorced	16	2	0
Thomas	male	48	secondary (vocational)	machine operator	single	12	2	0
Chloe	female	41	secondary (school-leaving examination)	office worker	divorced	18	3	1

*The National Institutes of Health Stroke Scale (NIHSS) is a standardized tool for measuring stroke severity or current stroke-related neurological deficits (the level of impairment caused by a stroke*). NIHSS has 15 components, which quantify individual components of the neurological examination such as consciousness, speech, motor, sensation, visual and cerebellar function, etc. The scoring range is 0 to 42 points, with higher numbers indicating greater severity. A score of <5 represents no stroke symptoms or a minor stroke, a score of 5 to 15 represents a moderate stroke, a score of 16 to 20 represents a moderate to severe stroke, and a score of 21 to 42 represents a severe stroke. All patients had a score of <5 at the time of the interview.

### Data collection

An in-depth, semi-structured interview format was employed to collect data. The research team developed the interview guide, encompassing various aspects of the illness trajectory chronology. A clinical neurologist was consulted during the guide development to ensure its relevance and comprehensiveness. The guide covered broad areas related to the illness progression, with questions focusing on the onset of IS, hospitalization experience, and subsequent quality of life after IS and return to pre-illness life. In addition, field notes were utilized to capture reflections on various elements, including the interview itself, the environment, the behaviour of the patient, and the interviewer’s self-reflection. The interviews took place between January and May 2023. The main interview questions were supplemented by prompts and probes that allowed a deeper insight into participants’ experiences. The three authors conducted semi-structured, face-to-face interviews (ŠŠ, LM, LM). The interviews were audio recorded and transcribed verbatim. The average interview length was 45 minutes, including a final debriefing conversation. The researchers conducted the interviews in the Czech language. Three participants were interviewed in a consulting room in the neurological outpatient clinic, and six were interviewed at home, according to their preferences.

### Data analysis

IPA analysed data using modified terminology (Smith et al., 2021). The IPA analytical process has seven phases: reflection; reading and re-reading; exploratory noting; constructing experiential statements; searching for connections across experiential statements; naming the personal experiential themes and consolidating and organizing them in a table; and continuing an individual analysis of other cases and working with personal experiential themes to develop group experiential themes (GETs) across cases (Smith et al., 2021).

The initial data analysis was conducted by the first author (ŠŠ) using NVivo software. The third author (LŠ) was involved in each step of the analytical process. The second (EG) and fifth (DB) authors assisted in conceptualizing the study and were involved in developing GETs across cases and interpreting the findings. After the analysis, an experienced researcher in IPA performed an independent audit. The IPA’s dual hermeneutic stance recognizes that the professional and personal experiences of the authors can lead to bias and preconceptions in the analysis process. Regarding positionality, two researchers (ŠŠ, DB) worked as nurses caring for patients after stroke, and three researchers (EG, LŠ, ŠŠ) were engaged in qualitative research on the quality of life and dignity of people with neurological diseases. Three authors are nurses; one author is a nurse and psychotherapist.

During the analysis, the authors discussed their annotations and emerging themes. This included reflection on biases and preconceptions arising from previous work and academic experiences. The transcripts were analysed separately by two researchers using the IPA idiographic approach. The final analysis for each case was established by agreement. Two teams of authors did cross-case analysis independently and then GETs, and final interpretation were developed based on consensus.

### Rigor

Trustworthiness and rigour of the research were ensured by implementing researcher triangulation, reflexivity, independent audit, and the research standard for qualitative studies COREQ.

### Ethics approval statement

Approval for the study was granted by the Ethics Committee of the University Hospital Olomouc and Faculty of Medicine and Dentistry, Palacký University Olomouc (Approval Number NU22-09 00,021, dated 6/2021). Before participating in the study, each participant provided informed consent, which assured their anonymity and confidentiality. To this end, the participants’ real names were not disclosed.

## Results

**Figure 2.**
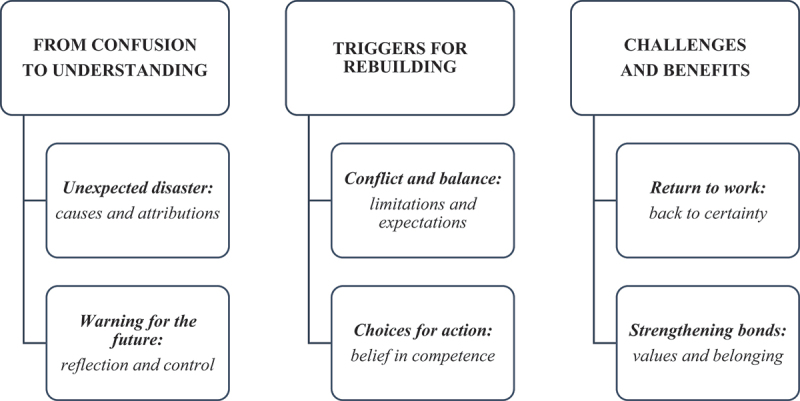
GETs and subthemes.

For the nine participants (Table I), IS represented an unexpected and sudden change in their lives, the consequences of which affected their quality of life and often altered their values for the future. Although a mild residual neurological deficit at enrolment was observed in all but two cases, the participants must make necessary lifestyle adjustments. Based on the participants’ experiences, three GETs and six subthemes were identified (Figure 2), representing changes in patients’ lived experiences after IS. Table II presents which themes emerged for each respondent.

**Table II.** Themes identified for each respondent.

GET/subthemes	John	Nina	Jack	Oliver	Emily	George	Sophie	Thomas	Chloe
**FROM CONFUSION TO UNDERSTANDING**									
Unexpected disaster: causes and attribution									
Warning for the future: reflection and control									
**TRIGGERS FOR REBUILDING**									
Conflict and balance: limitations and expectations									
Choices for action: belief in competence									
**CHALLENGES AND BENEFITS**									
Strengthening bonds: values and belonging									
Return to work: back to certainty.									

## From confusion to understanding

The importance that participants attribute to overcoming IS, as well as their emotional adjustment to having experienced it at a young age, has significant implications for their future lives. This theme is divided into two sub-themes: Unexpected disaster: causes and attribution; Warning for the future: reflection and control.

### Unexpected disaster: causes and attribution

For the participants, the sudden and unexpected onset of symptoms and their severity (impaired mobility, aphasia) was not only a shock but also a traumatic experience, a borderline situation in which they felt the loss of everything they had built and lived. At the time of hospitalization, they needed to understand the causes of their illness and wanted to know a clear prognosis, which the neurologists were unable to give them. They had a desire to communicate about their illness; some recall the absence of psychological intervention they required. For patients with aphasia, the communication problems aggravated psychological consequences.

*…  No way. Why me? Such a young girl. It was really awful. For me, at my age, it was a terrible disaster… Now that I look back on everything, it’s terrible because I’m forty-three, and I just can’t imagine having to just leave; I’ve got a husband, I’ve got a little baby. At that point, it was just like life was over for me because I was completely useless, mentally I was just completely down. (Emily)*

Most participants perceived that their stroke had been preceded by a period of accumulated stress at work or in a relationship and felt the need to find the “culprit” behind their illness. Understanding the cause of an own’s illness helps them develop strategies to cope with the situation and move on with their lives, sometimes even making important life decisions (e.g., changing one’s job, breaking up with a partner).

*…  I have it fixed in my mind that he [my partner] is the very reason why this happened to me, and I don’t think I will ever be able to accept that in my life. Internally, I feel that he had a huge part in it, and it’s like I can’t get over it. (Chloe)*

The consequences of IS, their extent and severity were seen as a result of a late diagnosis of the condition, which was manifested by constantly going back to the circumstances of the development of the problems and reassessing (*If they had intervened earlier…*).

*…  if I met the doctor who did the MRI, who could have maybe given a stronger push to just start acting faster, I probably would not scold him, but I would say to him: Man, it could have been done better there. Yeah, and there will be more of us, you know, or there will be others*. (George)

For some participants, surviving IS was also a catalyst for relationships. People around the responders, in their ignorance, were afraid to ask about their health and considered them disabled.

*…  and I felt like everybody was coming to see me like I was an animal in a zoo and it was terrible for me. That sometimes when I needed to say something, like… when I need to get a sentence out fast and there’s some stupid word, I just get stuck. (George)*

In the case of Oliver, hunters were surprised that he was able to keep his firearms licence; he sensed a turning away by his former friends and the stigma of stroke.

### Warning for the future: reflection and control

Some participants downplayed their stroke, seeing it as a minor illness that they had overcome and would not return to. They also did not acknowledge the seriousness of the illness but tended to trivialize it. Jack perceives his stroke as a matter of fate that he has no control over. He does not want to return to it, he pushes the experience out of his life:

*…  I’m such an easy-going guy, so I don’t even acknowledge these illnesses. It’s like it never happened, it’s just part of life, one time you have this, the next time you have that. I see it as something fated, so it happened, life goes on and everything is fine… So I don’t even accept the fact that I had some kind of stroke or whatever it was. I rather accept that I caught a disease and got away for 10 days. (Jack)*

An important factor influencing the degree of fear and anxiety about the future and the threat of recurrent stroke is the extent of residual neurological deficit. Minimal deficit and good clinical outcomes mean that participants are relieved and happy that things went well. They also appreciate family support in case of stroke recurrence.

*…  I was happy it turned out the way it did. That my consequences were actually so minor. Honestly, if things had gone badly for me, we would probably be talking to each other in a psychiatric ward, because I don’t know if I would have been strong enough to cope. The possibility of losing that job, losing that money as well, and yeah, actually, depending on the other person, I think that’s a terribly, terribly difficult fate for a person. I’m becoming more and more aware that things could have gone very badly for me. That I was actually a step away from, close to this. (Sophie)*

Despite the tendency of some patients to downplay or trivialize IS, the majority of respondents perceive the disease as a warning. They have changed their lifestyle, slowed down their pace of life, and are living more in the present moment. The experience of limitation and the feeling of vulnerability prompted participants to strive for greater self-control, a more responsible approach to life, or a reassessment of life’s priorities.

*… .so when I came back I just decided to take a different path… I re-evaluated my life after all that… I changed my job, found a new boyfriend… people I’m meeting now… a lot of new people, so… I guess that’s kind of boosted the confidence, my own confidence. (Chloe)*

On the other hand, John and Thomas identified themselves as “a biker” or “an athlete” to the extent that they do not want to radically change their lives, although they accept the health risks, they have reduced the risk factors (smoking, shift work at work) only partially. Both are single, without a partner and want to continue to devote themselves fully to their hobbies.

…  They wanted to admit me to the hospital right away, but I didn’t want to, I didn’t go until Monday. There was a birthday party, so I wanted to go to the birthday party. I would smoke some twenty-five, twenty, or twenty-five a day. And now I normally smoke six, seven cigarettes. I want to cut down a lot, not every day. I mean, I don’t feel any change. Except I think about it all the time, but nothing’s changed… But like I don’t want to change anything, you know, I’m only living once, like before, I’m not gonna sh** myself, right?. (Thomas)

## Triggers for rebuilding

After the stroke, the participants felt confused or lost in the context of the family and social responsibilities they would face in the future. Triggers for finding new meaningful perspectives in a situation included experienced marginal situations, feelings of vulnerability, and the need for help from others. Feelings of vulnerability manifested in fear of a recurrence of the stroke and loss of control.

During this time, it is very important to accept yourself as you are without comparing yourself to your pre-IS life. To overcome the consequences, it is necessary to know one’s limitations, but not to lose the will and desire to improve and manage.

### Conflict and balance: limitations and expectations

The degree of independence and functional status is important for the study participants who experienced a stroke at working age. At the time of the interview, no patient was dependent on another person for normal activities, except Nina, who required assistance in dealing with the authorities due to mild aphasia. In the absence of incentives to learn new skills and of support in follow-up services after hospital discharge, feelings of isolation and loss of confidence may grow in younger patients.

*I no longer have work challenges like before IS. Rather, I only have challenges to get myself in order as much as possible. I have it, but now I know that I just don’t have to have it here in an hour in half an hour. Yeah, but just fine, it will be in 2 months, but I won’t just make it worse, because of course, the more I get under some pressure, the faster my body reacts to it. (George)*

*…  Well, I was able to accept, like [stammering], I used, I used to speak Greek, I used to speak English, but those languages, I dropped Greek completely and now I can’t speak any, not even English. I used to speak English, now I speak a little bit of English, but that’s all… My mother used to come with me everywhere… but I was just nodding. (Nina)*

Resuming leisure activities, sports, and hobbies was also a strong motivation for the participants.

*…  I would like to at least go on. I used to ride a motorbike, you know. I would love to get back on a motorbike, but you just have to make the handwork, at least in terms of sensitivity and grip, just with the brake or the clutch. So that at least this basic movement is kind of well controlled, and it’s not right now. So just that interest in the sport, at least I could keep the bike. (George)*

### Choices for action: belief in competence

It is not only time that plays a role in the road to recovery, but also coping strategies that proved helpful to the participants in the past. A significant “driver” was their partner or children.

*…  when my daughter saw me, she got very emotional, and then she cried on the phone and told me that she wanted to have back the mommy that was taken away. And I think that’s what really, what spurred me on to decide that I wasn’t going to give up.” (Chloe)*

Sport as a life philosophy, the motivation to fight and win as in sports, provided another means of better coping. Participation in activities related to rehabilitation and recovery increased the participant’s sense of competence and meaningfulness as rehabilitation goals were progressively achieved. Getting better and progressing in rehabilitation led to experiencing positive emotions (joy, hope, gratitude, confidence, and satisfaction) that contributed to recovery and a return as close as possible to the original active lifestyle.

*…  the improvement from not being able to get on one’s feet and then being able to get on one’s feet, it’s just huge, it’s very, incredibly enjoyable… It was easier for them to work with me and take it a little bit further again. And that every little step is just a victory for me. (George)*

*… On the second, and third day in the hospital, the doctor came to see me about rehabilitation. So she walked me along the corridor, we went up the stairs, I ran up two flights of stairs, and then I had to wait for her to catch up with me, right? I never thought about not being able to do something physically. I tried everything. I’m an active athlete, I was in a very good physical shape. I’ve been running a sports club for 25 years, it’s my life’s work. I have 75 year old guys there. So, if they can do it, I just don’t have the right to just say I can’t (John)*

Another important factor is the motivation and support provided by loved ones. They replace formal forms of social support, which are often neglected, underestimated, or overlooked by participants.

*…  it takes time, it just needs willpower, it just needs a healthy environment, healthy people around you who love you, and you sort of have to struggle along. Just exercising and training your brain… really just trying to get out, going for walks, just being around people. Drinking lots of fluids, reading and just being with friends and loved ones. And especially finding that positive energy inside of you. (Emily)*

## Challenges and benefits

Surviving IS was describes as a significant turning point in a participant´s life; in addition to several negative effects and changes, it can also mean changes with positive consequences and perceived benefits for them. Many patients have reassessed their relationships and priorities; some have chosen to make major life changes that they perceive as positive. They often set boundaries (learning to say no, taking a break) and gain new skills that help them regain control of their lives and accept new constraints.

### Return to work: back to certainty

Returning to work is a fundamental demonstration of the possibility of resuming one’s former life as the participants described. It is important that employers are interested in enabling participants to return to work after recovery from IS. Some employers were very negative about returning to work, doubting the participant’s ability to do so, even though they had accepted the participant’s return to work.

In other cases, they allowed reduced working hours or offered a different position; in some cases, there was a complete job change. Work is an affirmation of participant’s worthiness and a source of self-fulfilment, and it also functions as cognitive training.

*… ”The tolerance of the people around me is much greater here than in my previous job, and that helps me a lot… After the stroke, of course, they gave me a break, yes. During that year. But now I’m trying to get back on track. If I had that stroke in my old job, I’d probably have to quit right away. Because even after that month, or I mean even after six months, it would be impossible to go back because pulling that weight is too much, it’s just impossible. You need to have either a part-time job or just a job where you can make your own schedule. Or where they meet you halfway. Yeah, that’s good. (Sophie)*

George described returning to work as a form of “mental rest”:

*… ”The work, because it just has its own order, its own rules, and its own clearly defined things that can’t be done any other way, so you like to go back to the certainty that this is how the work is done, and this is how it should be*. (George)

However, the opposite effect can also occur, whereby John asked for an exception to be able to return to shift and night work. The employers’ approach impacts the participants’ mental state, their socialization, and, finally, their financial security.

*…  The doctor was the one who gave me the most trouble. I immediately got caught up in charts and a ban on night shifts. That’s the best shift; night shift is just fine*. *(John)*

### Strengthening bonds: values and belonging

Experiences with IS have shown that family and friends are the most important sources of support. The family was described by participants as the cornerstone of support as well as motivation. Thanks to this support, it is possible to observe positive consequences based on testimony of participants: greater self-care, greater care by family members for each other, a closer relationship with one’s partner, a more relaxed pace of life, and increased self-esteem. The experience of IS itself also has an impact on family members as some have stated. For example, the children’s school performance may deteriorate, or the spouse may worry about their partner and the family’s overall functioning in the future. Family members of participants usually try individually to find a way out of the crisis that IS undoubtedly causes and to support each other. In the case of partners living in a stable union for a long time, the relationship can be strengthened and strengthened even more after overcoming IS and supporting the partner.

*…  I say every cloud has a silver lining, so I can see that I just have this supportive environment there. If things got really bad, they would kind of function… You could see that they liked me, so of course it made me very happy to have that backing here as well. That’s why I say that my life is much better now than it was before the* stroke.*(Sophie)*

Support from loved ones and health professionals took various forms for them: providing help, encouragement, and motivation, sharing experiences, a sense of belonging, and assistance in acquiring new skills.

*…  That kind of moral support or psychological support from that nursing staff or those physical therapists was huge. So the hope was there for me that I would be back to at least 80% within a year.* (*George*)

## Discussion

Evidence on patient-reported outcomes and experiences of young adult patients after ischaemic stroke is limited. The present study utilized IPA to explore how young adults perceive the meaning of IS over 12 months after its onset. By conducting an IPA, three themes were identified that should be considered in line with supporting patients and their quality of life: from confusion to understanding, triggers for rebuilding, and challenges and benefits. We found that young stroke patients, more than 12 months post-stroke, move from confusion to understanding. In addition, the experience of limitations and belief in competence were identified as the triggers to the process of meaning perspective transformation (Kessler et al., 2009). Our findings indicate that even minor neurological impairment after IS negatively impacted quality of life, as some reported recently in quantitative studies (Boudokhane et al., 2021; Yoon et al., 2021). We identified specific challenges and benefits that young stroke patients face during long-term post-stroke care and their decision-making process of returning to the previous pre-stroke life. Among these were returning to work and strengthening family relationships. The changes occurring following IS at a young age can be explored through transformative learning theory (Kessler et al., 2009). This theory can be utilized as a framework to explain adjusting to post-stroke sequels because, through critical reflection, a person in this disabling life situation can reassess the assumptions on which beliefs are based and gain new insights on which to base his or her actions (Kessler et al., 2009). Transformative learning has been considered important in gaining insight into how stroke survivors can learn, rebuild competence, and re-engage in valued activities. The grounded theory approach was applied to explore lived experience following stroke and changes in meaning perspective among 12 stroke survivors at least one year after stroke. The process of meaning perspective transformation following a stroke based on transformative learning was presented in this qualitative study. This process includes the interaction of four factors contributing to transformation: triggers, support, knowledge, and choices to action (Kessler et al., 2009).

The experiences of young adult patients after IS often focus on recovery from functional impairment (e.g., Arntzen et al., 2015; Kitzmuller et al., 2013). Many qualitative studies describe the impact of IS primarily in terms of perceptions of one’s body and feelings of difference from others (Immenschuh, 2003; Lawrence & Kinn, 2012), and stigma has been frequently reported in young adult patients after IS (Hanney, 2012; Holloway et al., 2022; Immenschuh, 2003; Leahy et al., 2016; Wolfenden & Grace, 2015). In those studies, stroke-related stigma stemmed from IS being stereotyped as a disease relevant to older adults. This is also why the studies identified themes reflecting experiences with a delayed diagnosis or misdiagnosis. The stigma of a stroke at a young age is associated with breaking the unwritten rule of being “young, healthy, and productive” and subsequent fear of social exclusion (Immenschuh, 2003). Therefore, other people’s reactions were assessed in light of this feeling of abnormality.

Ontological certainty is often disrupted in IS survivors (Alaszewski et al., 2006, which is associated with greater vulnerability in these individuals (Wolfenden & Grace, 2015). For the participants in this study, these experiences, limitations, and feelings of vulnerability threatened their sense of control over their own lives). The meaning of IS at a young age can be described as an unexpected transition (unpredictable and stressful life events) in an adult’s life (Tønseth, 2018). Some participants reported changes after IS as triggers (turning points), giving their lives a new meaning and direction.

One of the main factors of the existing uncertainty is the change from the previous hectic and diverse working-age life caused by an unexpected and atypical event of IS (Brouns et al., 2019; Kessler et al., 2009). Such a disruption can be found in our first theme, which progresses from confusion to understanding. The theme of disorientation was also identified in a study by Lawrence (2010). Sudden and unexpected IS also often prompts patients to seek responsibility for their condition, as reflected in the second subtheme of reflection and control. In this context, downplaying the condition, fear of recurrence, and not admitting to IS were described. Feelings of uncertainty about the trajectory of recovery and possible consequences are also frequently described in the literature (McKevitt et al., 2004). These states may also be triggered by the reported trivialization by healthcare professionals or the possible absence of patient-centred care appropriate for patients’ age and milder disability, as documented by some authors (Holloway et al., 2022; Röding et al., 2003).

Experiences of limitation, vulnerability, and the need for help often lead young adults to triggers for rebuilding. Loss of activities, skills, and independence, as well as emotional and social losses described in the literature (McKevitt et al., 2004; Salter et al., 2008; Satink et al., 2013), are related to internal conflicts, as seen in our sample. However, in addition to loss, feelings of insecurity, social isolation, adaptation, and reconciliation, themes of change, transition, and transformation were also described in a meta-synthesis of qualitative studies of young adult IS patients (Salter et al., 2008); the themes related not only to balancing on the edge of possible expectations, but also to coping strategies. Among these, family, sport, rehabilitation, and support from loved ones were particularly reflected. Satink et al. (2013) describe needs related to self-management support, information, psychological, emotional, and social support. A disturbed sense of self is typical for young adults (Immenschuh, 2003; Lawrence, 2010). Its impact is also associated with self-continuity and self-growth (Hutton & Ownsworth, 2019). Young adults’ experiences of IS often arise from Bury’s model of chronic illness (Bury, 1982; Immenschuh, 2003; Kuluski et al., 2014), but equally important are choices for action. Similarly, Kitzmuller et al. (2013) found that individuals seek to restore their sense of self.

In terms of challenges and benefits, new skills are important, helping to accept the limitations brought about by IS. Return to work (RTW) is very important for young adults, as is strengthening attachments. Isolation and change from the “former self” are major social consequences (Morris, 2011). Often invisible impairments may not always be well understood in social and work contexts (Wolfenden & Grace, 2015), returning to the fact that the psycho-emotional and cognitive needs associated with downplaying not only in healthcare settings may be poorly understood by those around (Shipley et al., 2020). Closer relationships with family, including the need to “belong somewhere,” stronger attachments, and deeper appreciation, are described as much needed (Hanney, 2012; Kuluski et al., 2014). Socialization regarding RTW tends to be a bridge to recovery for young adult IS patients (Edwards et al., 2018). La Torre et al. (2022) document that RTW may also warrant higher self-esteem and life satisfaction levels. According to a qualitative meta-synthesis (Frostad Liaset & Lorås, 2016), empowerment, self-awareness, motivation, and facilitation are four key concepts within the RTW process after acquired brain injury.

The described devaluation of the social construct, the acquisition of physical/cognitive impairment in terms of impaired quality of life, as well as the persistent lack of awareness of the prevalence of IS at a younger age (Shipley et al., 2020) underscore the need to monitor further young IS adults’ experiences and to collate the results. Focusing on issues that need attention in young adults may contribute not only to designing effective interventions but also to improving the quality of life of these individuals, including alerting others to the possible downplaying of the condition at young age. Further studies are needed to identify new strategies that may help to improve and accelerate recovery in young adults with IS.

## Limitations

The article is based on the results of a small qualitative study carried out in a single cerebrovascular centre in the Czech Republic. Yet the findings contribute to our understanding of the experiences reported one year after IS by young adults with no or minimal residual deficit. As the participants were interviewed at different phases of their post-IS rehabilitation and recovery, longitudinal research on adaptation to stroke should follow. The small participant group and recruitment from one treatment centre limit the ability to generalize the results. The study was not designed as a longitudinal study, which allowed a focus on the long-term experience following a stroke at home but did limit the ability to see how experiences change over time. Another area for improvement of the study is the relative shortness of the interviews. This may be attributed to fatigue, a typical feature of the condition. Rather unusually, four co-authors were involved in the analysis and interpretation process. Our strategy was motivated by an effort to increase the credibility of the process by incorporating diverse perspectives that enhance the final interpretation.

## Conclusion

The paper contributes to the young stroke literature in the Central European context to identify the current gaps and limitations in supporting the needs of stroke patients of working age in long-term post-stroke care. Three group experiential themes were identified: from confusion to understanding, triggers for rebuilding, and challenges and benefits. We found that stroke patients of working age, more than 12 months post-stroke, move from confusion to understanding. The experience of limitations and belief in competence were identified as the triggers to the process of meaning perspective transformation. The current findings indicate that even minor neurological impairment after IS negatively impacted patients’ lives. Specific challenges and benefits that young stroke patients face during long-term post-stroke care and their decision-making process of returning to the previous pre-stroke life. Among these were returning to work and strengthening family relationships. The study helps to understand the experiences and to develop new interventions specific to this age group. These findings are crucial for healthcare professionals to develop improved age- and mild- impairment-appropriate strategies or tailor self-management interventions for stroke patients of working age.
